# 

**Published:** 1916-09

**Authors:** 


### Publishers' Announcement

Now that the summer is over and vacations past, the dental world is settling down to its work and making enormous demands for supplies upon the manufacturers and dealers. More dentistry will be done than ever before and the demands upon the dentists will be more than customary.

War times have brought many to our shores and have kept many here and this, together with the usual increase in population, makes an unusual increase. People know more about the advantage in a preventive way, of having dentistry done, and tooth brush drills are teaching the youngsters the value of dentistry also. Social life, marriages, receptions, clubs, golf, motoring, athletics, all call attention to the teeth and have influence on having dental work attended to. Greater earnings, higher wages, better social surroundings, eight hour days, shorter business hours, all facilitate the dental work. There is plenty of work for everyone.

The Dental Register will have an unusual interesting run of scientific articles this winter, and we trust all subscribers will send in renewals of their subscriptions as early as possible. The fall campaign for subscriptions will soon commence and subscription blanks will be sent as usual. Dental supply houses everywhere will receive your subscription to the Dental Register and forward it to the publishers.

